# New Chloroplast Microsatellites in *Helichrysum italicum* (Roth) G. Don: Their Characterization and Application for the Evaluation of Genetic Resources

**DOI:** 10.3390/plants13192740

**Published:** 2024-09-30

**Authors:** Matjaž Hladnik, Alenka Baruca Arbeiter, Petra Gabrovšek, Félix Tomi, Marc Gibernau, Slavko Brana, Dunja Bandelj

**Affiliations:** 1 Faculty of Mathematics, Natural Sciences and Information Technologies, University of Primorska, 6000 Koper, Slovenia; matjaz.hladnik@upr.si (M.H.); alenka.arbeiter@upr.si (A.B.A.); petra.gabrovsek@famnit.upr.si (P.G.); 2 Laboratoire Sciences Pour l’Environnement, Université de Corse-CNRS, UMR 6134 SPE, Route des Sanguinaires, 20000 Ajaccio, France; tomi_f@univ-corse.fr (F.T.); gibernau_m@univ-corse.fr (M.G.); 3 Istrian Botanical Society, Trgovačka 45, HR-52215 Vodnjan, Croatia; istra.botanica@gmail.com

**Keywords:** immortelle, chloroplast genome, microsatellite markers, allele sequencing, genetic diversity, haplotypes

## Abstract

*Helichrysum italicum* (Roth) G. Don is a Mediterranean medicinal plant with great potential in the cosmetics, culinary and pharmaceutical fields due to its unique bioactive compounds. Its recent introduction into agroecosystems has enhanced the exploitation of genetic diversity in natural populations, although limited molecular markers have made this challenging. In the present study, primers were designed for all 43 SSRs (72.1% mononucleotide, 21% dinucleotide and 6.9% trinucleotide repeats) identified in the chloroplast genome. Populations from Cape Kamenjak (Croatia) and Corsica (France) were analyzed with ten carefully selected cpSSR markers. From the initial set of 16 cpSSRs amplified in all samples, 6 cpSSR markers were removed due to low-length polymorphisms, size homoplasy and nucleotide polymorphisms that could not be detected with allele length. Of the 38 haplotypes detected, 32 were unique to their geographic origin. The highest number of private haplotypes was observed in the Cape Kamenjak population (seven out of nine detected). Based on clustering analyses, the Kamenjak population was the most similar to the Capo Pertusato (south Corsica) population, although only one sub-haplotype was shared. Other Corsican populations were more similar to each other. A cross-species transferability test with *Helichrysum litoreum* Guss. and *Helichrysum arenarium* (L.) Moench was successfully conducted and private alleles were identified.

## 1. Introduction

The immortelle (*Helichrysum italicum* (Roth) G. Don) is an aromatic plant of the Asteraceae family widely distributed in the natural vegetation of the central Mediterranean Basin. This plant is appreciated for its essential oil and other plant extracts possessing high-valued, biological active compounds used in the pharmaceutical, cosmetic, perfumery and food industries. Several studies have reported and confirmed its antimicrobial, anti-inflammatory, antioxidant, anti-viral, anticancer, insecticidal, anti-aging and repellent activities [1,2,3,4,5]. These beneficial properties of immortelle extracts have led to the increased interest in its cultivation and domestication in some Mediterranean countries, and opened the need for a more accurate characterization of its genetic resources.

The taxonomy of *H. italicum* is complicated and was revised several times in the past [6,7]. The most recent multidisciplinary revision of this species divided it into four subspecies (subsp. *italicum, tyrrhenicum, microphyllum* (Willd.), *siculum* (Jord. and Fourr.)) distributed through different regions of the Mediterranean Basin [8]. Their morphological features and geographical distribution are well described by Galbany-Casals et al. [7] and Herrando-Moraira et al. [8]. It should be noted that within populations from a small geographical area, a high diversity of individual plants has been observed [9], which is accompanied by a remarkable phenotypic plasticity that also leads to different chemotypes, mostly as a result of adaptation to diverse ecological factors in the habitats in which they grow and also due to distinct genetics [7,10]. For example, the essential oil of Tyrrhenian immortelle is characterized by a high content of neryl esters, the most distinctive constituents valued in the perfumery industry and aromatherapy. Essential oils from the Balkan and Adriatic coast, on the other hand, contain mainly pinenes and a high curcumene content (reviewed by Ninčević et al. [5]). Thus, the essential oil from the Corsican origin reaches a higher price on the market [2]. Therefore, it can be expected that some countries will decide to develop the quality certification Protected Geographical Indication (PGI) to prevent uncontrolled exportation of the plant from their native growing sites and to protect the quality/typicality of immortelle essential oil. These initiatives will probably contribute to the stricter control of the planting material offered on the market, and to a higher price of raw material.

Natural growing sites of some subspecies and species overlap, and their spontaneous hybridization has created new hybrids with intermediate morphological characters [7]. An example of this are hybrids between *Helichrysum litoreum* Guss. (avowed substitute for *Helichrysum angustifolium* (Lam.) DC [11,12,13]) and *H. italicum* subsp. *italicum.* These two species share natural habitats in west–central Italy, some islands in the Tyrrhenian sea and the Croatian Istra Peninsula [7].

Plant genetic resources used for the production of plants for plantations are collected from seeds in natural populations. However, the morphological diversity and chemotype variation in *H. italicum* make it difficult to accurately identify plant species and determine the most promising plants for cultivation based on morphological traits and chemical analyses only. The observed differentiations of chemotypes are especially interesting for a diversified industrial use of the plant [10,14]. In addition, the high price of immortelle essential oil on the market contributes to its intentional adulteration, and the gap between availability and demand for immortelle essential oil is often filled with plant material from other species of questionable chemotype and origin [2]. Adulteration is the main problem for both the industry and consumers, as it jeopardizes the quality of immortelle products. Therefore, mechanisms for the identification and traceability of genetic resources on the market are necessary.

The characterization of plant genetic resources with molecular markers is therefore important not only for material traceability but also (a) to understand genetic diversity within and between populations from geographically distinct regions with defined ecological factors for the development of a systematic plan for field examination; (b) for breeding programs, for the selection or development of cultivars with superior traits and also for the controlled propagation of planting material with high performance for industrial use; and (c) to establish a solid basis for further studies to link genotypes to the chemical composition of extracts or metabolome diversity and, consequently, to pharmacology.

In comparison to other aromatic plants, the genomic research of immortelle is lagging far behind. Only recently have the first twenty-four nuclear microsatellite markers been developed from genomic sequences of the Adriatic immortelle [12]. However, the first genomic sequences, obtained with high-throughput sequencing technology, enabled the assembly of the immortelle chloroplast genome [15] and provided the opportunity to develop new chloroplast-based markers.

Sequence analyses of chloroplast genomes have contributed significantly to the phylogenetic studies of several plant families and revealed considerable variation within and between plant species [16,17], providing a valuable source for genetic marker development. It should be noted that, since photosynthesis is one of the pivotal mechanisms for plant biomass accumulation, organellar genome editing offers additional opportunities for plant breeding [18].

Traditionally, for the authentication and identification of plant barcodes, *matK* and *rbcL* from two chloroplast coding regions have been suggested as standard markers [19], which could additionally be supported with other barcodes, such as *trnH*-*psbA*, plastid intergenic spacer region and nuclear ribosomal ITS [20]. The chloroplast intergenic spacer *psbA*-*trnH* was used for the characterization of *H. italicum* and other closely related species, but considerable sharing of haplotypes among taxa was observed [6].

Chloroplast SSRs (cpSSRs) have been recognized as powerful markers due to their polymorphism and ability to detect intraspecific diversity, as well as the fact that they inherit the characteristics of the chloroplast genome, such as uniparental inheritance, non-recombinant nature and haploid state [21]. The latter could provide information about the dispersal of seeds (if chloroplasts are inherited maternally) or pollen (if they are inherited paternally). In addition, cpSSRs are more effective indicators for population subdivision and differentiation compared to nuclear markers, and the combination of cpSSRs with nuclear SSRs can further enrich various genetic studies [22].

The present study focuses on the development of new cpSSR markers derived from the sequenced chloroplast genome of *H. italicum*. The newly developed markers were characterized in different immortelle populations from Corsica and the northeastern Adriatic coast, which represent important genetic resources for essential oil production and transfer of plants to agricultural cultivation. The transfer of wild populations from nature to the agroecosystem and further industrial utilization require the control of the origin of the planting material, which is why new cpSSRs represent an important molecular tool. The usefulness of cpSSRs for the unambiguous differentiation of three *Helichrysum* species (*H. italicum*, *H. litoreum*, *H. arenarium* (L.) Moench) was also tested.

## 2. Results

### 2.1. Chloroplast Microsatellites Characterization

**Figure 1:**
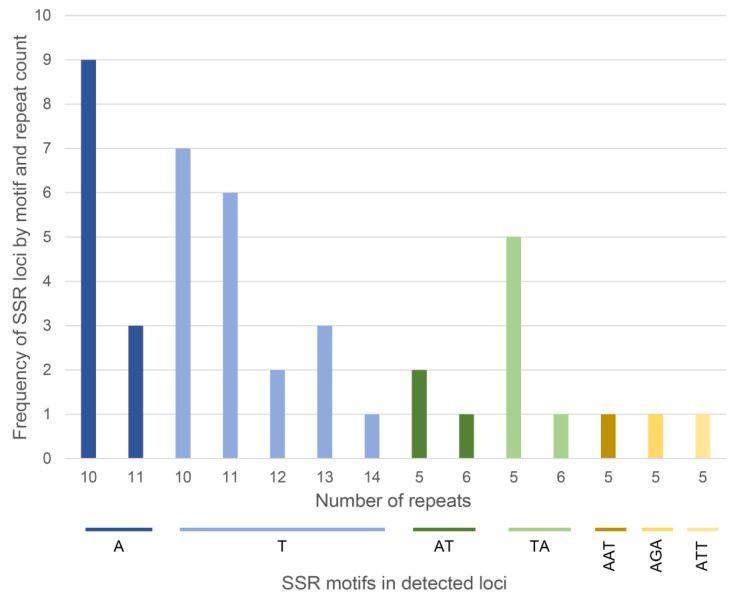
Overall abundance of various SSR repeat types and number of repeat units in *H. italicum* chloroplast genome. Note. An example interpretation of the first bar: Nine SSR loci with the A motif contain 10 repeats.

In the present study, the complete chloroplast genome sequence of *H. italicum* [15] was examined with the MISA tool for microsatellite identification. In total, 43 SSRs were identified. These SSRs comprised 31 mononucleotide repeats (72.1%), 9 dinucleotide repeats (21.0%) and 3 trinucleotide repeats (6.9%) (Figure 1). Notably, there were no tetranucleotide, pentanucleotide or hexanucleotide repeats. Mononucleotides consisted exclusively of the nucleotides A or T. The most frequent mononucleotide motif was T, which accounted for 44.2% (of all identified SSRs), followed by the A (27.9%) motif. Among dinucleotides, the TA motif (14.0%) was the most abundant, followed by the AT (7.0%) motif. From the perspective of SSR distribution, 34 SSRs (79.1%) were observed in the LSC (long single copy) region, 7 SSRs (16.3%) were observed in the SSC (small single copy) region and 2 SSRs, cpHiUP-43 and cpHi-UP-38, were found in the IRA (inverted repeat A) and IRB (inverted repeat B) regions, respectively. The SSR motif from locus cpHiUP-38 starts at the end of the LSC region, but the majority of it is located in the IRA region. Upon examining the complementary regions of the last two SSRs (alignment of cpHiUP-43 against IRB region and cpHiUP-38 against IRA region), it was revealed that their complementary regions had only 9 repeats of T, whereas the detected SSRs had 10 repeats of T (with 10 being the threshold for the MISA tool) and thus were not detected. However, the flanking regions were identical in the complementary region of cpHiUP-43 and one additional SNP was observed in the complementary region of cpHiUP-38.

In addition, the results showed that most SSR repeats were distributed in the intergenic regions (28 SSRs; 65.1%) as they always contain more polymorphisms, followed by the protein-coding and non-coding RNA genes (15 SSRs; 34.9%), including *trnK-UUU*, *rpoB*, *rpoC1*, *rpoC2*, *ycf1* and *ycf3* genes, while no SSR repeat was observed in rRNA genes.

### 2.2. Primer Design and Marker Validation

Primers were designed for all SSRs. Test amplification was performed with four samples from different *H. italicum* populations (Kamenjak, Capo Pertusato, Cavu and Col de Bavella) to increase the probability for detecting different alleles. DNA from an individual *H. italicum* plant (Črišnjeva, Croatia), previously collected for high-throughput sequencing [12] and chloroplast genome assembly [15], was used as a reference sample for the PCR amplification of all tested loci. Finally, 16 SSRs (all 12 di- and trinucleotide repeats, and the 4 mononucleotide repeats presenting the highest polymorphism) were selected for further analysis of the ten *H. italicum* populations (194 samples) obtained from Cape Kamenjak (Istrian peninsula, Croatia) and the island of Corsica (France). The final selected set of SSRs with primers and amplicon lengths, observed in the sequenced *H. italicum* plant, is listed in Table 1, whereas data related to the rest of the mononucleotide SSRs are detailed in Table S1.

All 16 microsatellite markers amplified successfully across the ten *H. italicum* populations and the allele size ranges at all loci were comparable with expected allele sizes (Table 1). However, cpHiUP-02, cpHiUP-03 and cpHiUP-11 were monomorphic, although sequencing of the cpHiUP-03 alleles revealed the presence of a SNP in a few samples (Figure S1). All three mentioned loci were excluded from the population analysis. Additionally, two alleles with the same length but with different nucleotide sequences were identified in the locus cpHiUP-04, making this locus unsuitable for fragment analysis. The SNPs also observed in some cpHiUP-09 alleles with the same length and the low discrimination ability of *H. italicum* samples of locus cpHiUP-10 (only one sample had allele 207, whereas in all other samples, allele 213 was observed) were also reasons for excluding these two cpSSR loci. Finally, out of the 12 di- and trinucleotide SSR loci, 6 loci (cpHiUP-01, cpHiUP-05, cpHiUP-06, cpHiUP-07, cpHiUP-08 and cpHiUP-12) were selected for further analysis. Contrary to di- and trinucleotide SSRs, all four mononucleotide SSRs were kept in the final dataset for population analysis.

The source of length polymorphism was due to a different number of SSR motif replications in all finally selected di- and trinucleotide SSRs, but some alleles showed mutations also in flanking regions. Allele 252 of the cpHiUP-05 locus showed duplication of the 18 bp region present within the TA motif (Figure S1). Locus cpHiUP-07 showed the highest variability. Alleles 291 and 293 of the cpHiUP-07 locus were different because of different numbers of SSR repeat motifs, whereas 285, 287 and 297 alleles were different because of the insertion of two thymines in the mononucleotide region (287), duplication of the ATTAAT motif (297) and duplication of the TCTAT motif (297). All three alleles had a deletion of an ATAAATAT sequence compared to the 291 and 293 alleles. Similarly, the cpHiUP-12 218 allele had a duplicated 15 bp motif (TTATATTCAATCGTC), whereas allele 187 had a deletion of an AAATAGAA motif in a flanking region (two such motifs were present in other alleles). Due to difficulties in sequencing loci with mononucleotides, the source of polymorphism could not be detected for all alleles despite several attempts. However, based on the successfully sequenced alleles and the observation that most alleles differ in length by 1 bp (with some exceptions), all four mononucleotide cpSSRs were included in the genotyping analysis.

In total, 38 alleles were detected in 10 chloroplast SSR loci among the 194 samples. The number of amplified alleles (N_a_) at each locus ranged from 2 (cpHiUP-05, cpHiUP-08) to 7 (cpHiUP-15), with an average of 3.8 alleles. The effective number of alleles (N_e_) ranged from 1.03 (cpHiUP-08) to 2.05 (cpHiUP-14), with an average of 1.49. Shannon’s information index (I) varied between 0.080 (cpHiUP-08) and 0.974 (cpHiUP-15), with an average value of 0.516, whereas the observed diversity by locus (h) was between 0.030 (cpHiUP-08) and 0.513 (cpHiUP-14), with an average value of 0.271 (Table 2).

### 2.3. Genetic Structure of H. italicum Populations

The genetic diversity parameters of the studied *H. italicum* populations are summarized in Table 3. The highest number of alleles (from all ten cpSSR loci) was obtained in the Kamenjak population (25) and the lowest in Sisco (14), representing 66% and 37% of all 38 alleles, respectively. Of the ten cpSSR markers analyzed, seven produced 10 private alleles for seven populations (Table 4). The highest number of private alleles (three) was characteristic for the cpSSR marker cpHiUP-15, and only one private allele was detected at seven loci (cpHiUP-01, cpHiUP-06, cpHiUP-07, cpHiUP-08, cpHiUP-12, cpHiUP-13 and cpHiUP-16). Two of the private alleles were observed for Kamenjak, Pertusato and Corte, and one private allele for Col de Bavella, Punta di a Vacca Morta, Col de Saint-Eustache and Ajaccio. No private alleles were detected for the populations in Cavu, Lavu Santu and Sisco (Table 3). The Shannon’s information index across populations ranged from 0.169 (Sisco) to 0.665 (Punta di a Vacca Morta), with an average value of 0.309.

**Figure 2:**
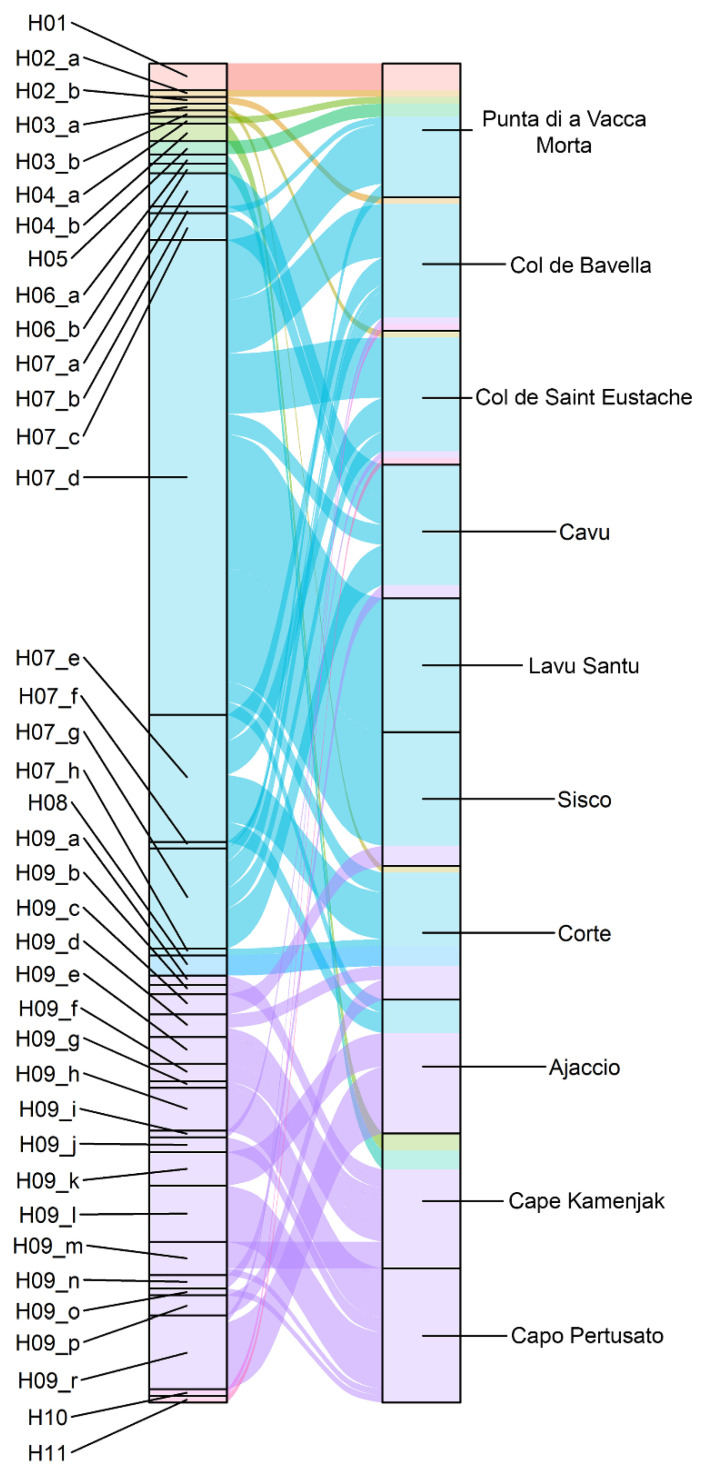
Distribution of haplotypes and their sub-haplotypes across populations, all from Corsica, except Kamenjak (Croatia). Haplotypes, as determined with di- and trinucleotide SSRs, are marked with different colors, whereas sub-haplotypes are indicated by a horizontal line within each haplotype.

Based on the observed genotyping data for all *H. italicum* samples (and eight *H. litoreum* samples that served as the outgroup species), the haplotypes and their distribution across populations were determined. Eleven haplotypes were identified when only six di- and trinucleotide SSRs were taken into account (and an additional six haplotypes were determined for *H. litoreum*), whereas thirty-eight haplotypes were identified in *H. italicum* samples with four additional mononucleotide SSRs. To visualize the differences between haplotypes based on six di- and trinucleotide SSRs and four additional mononucleotide SSRs, the main groups of haplotypes were labeled as H01 to H17, while further divisions based on mononucleotide SSRs were labeled as sub-haplotypes (e.g., H02_a, H02_b, etc.) (Figure 2 and Figure S2). Haplotypes H07 and H09 had the largest number of sub-haplotypes (8 and 17, respectively), while haplotypes H01, H05, H08, H10 and H11 were not further divided in sub-haplotypes. The *H. litoreum* samples showed species-specific haplotypes (H12, H13, H14, H15, H16 and H17). Two haplotypes, H07 (in blue) and H09 (in violet), were the most highly represented across the sampled locations: Ajaccio, Pertusato and Kamenjak predominantly featured the H09 sub-haplotypes, while H07 sub-haplotypes dominated in all other populations (Figure 2 and Figure S2).

The number of haplotypes per population (A) ranged from one to nine, and the effective number of haplotypes (N_e_) was between 1.00 and 7.26. When all populations were analyzed together, the mean A and mean N_e_ were 5.40 and 3.63, respectively. With the exception of the Lavu Santu population, in which only one haplotype was found, the values for haplotypic richness (R_h_) ranged from 0.99 (Sisco) to 8.00 (Kamenjak), with a mean value of 4.00. Similarly, genetic diversity values (H_e_) were between 0.268 (Sisco) and 0.924 (Kamenjak), and most populations had a genetic diversity value above 0.700. The mean genetic distance (D^2^_sh_) between individuals varied more widely, ranging from 0.524 (Cavu) to 86.404 (Punta di a Vacca Morta), with a mean value of 15.310 (Table 3).

Out of 38 haplotypes, 32 private haplotypes (P) were found in nine out of ten *H. italicum* populations (Table S2). The highest number of private haplotypes was found in Kamenjak (seven), followed by the populations of Pertusato (five), Punta di a Vacca Morta (five) and Corte (four), while three private haplotypes were found in Cavu, Col de Bavella and Col de Saint-Eustache, and only one in Ajaccio and Sisco. The frequency of the private haplotypes was mostly between 0.05 and 0.25, with the exception of the two haplotypes (H09_h and H09_l) in the Pertusato population, which had a frequency of 0.32 and 0.42, respectively.

Gene diversity within populations (H_s_) ranged from 0.026 in the Sisco population to 0.082 in the Corte population (Table 5). The values of D_st_ (diversity due to differentiation) were lower than H_s_, ranging from 0.014 to 0.034, indicating that most of the genetic diversity was found within populations. In order to assess the level of genetic differentiation among the *H. italicum* populations, the F_st_ values (proportion of the total genetic differentiation) between populations were calculated. The highest difference was found between the populations of Lavu Santu and Pertusato (F_st_ = 0.185; data not shown) and the lowest difference between Sisco and Lavu Santu (F_st_ = 0.010; data not shown). As expected, the proportion of total genetic differentiation within populations was higher than between populations. In addition, the relatively low overall value of F_st_ (0.266) indicates that a low to marginal proportion of diversity (26.6%) is observed between populations.

**Figure 3:**
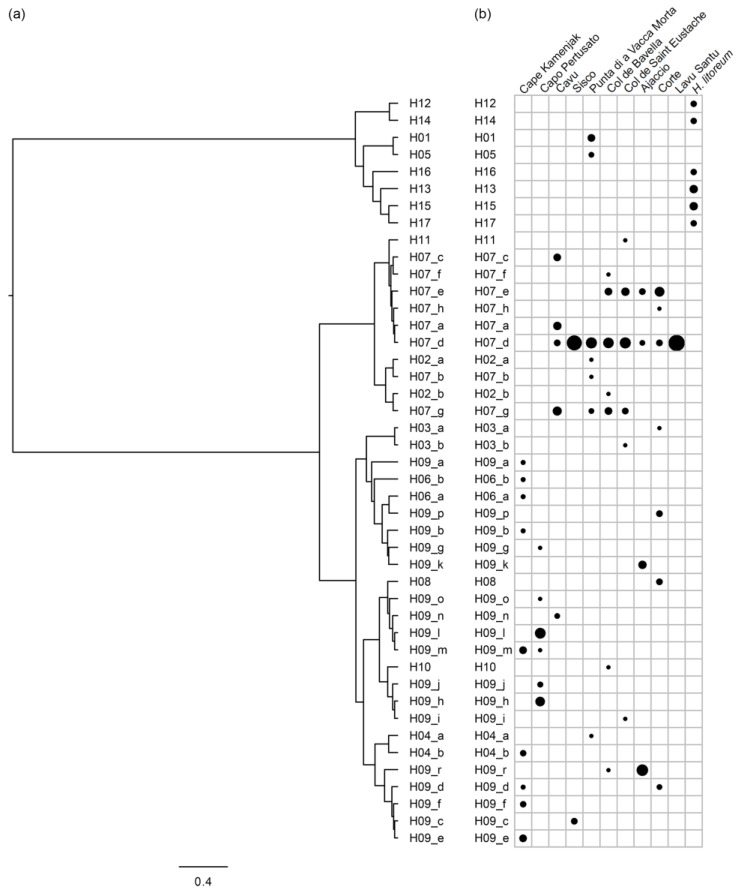
(**a**) Dendrogram with *H. italicum* and *H. litoreum* (sub-)haplotypes based on Bruvo’s distance and Ward’s linkage method for hierarchical clustering. (**b**) Frequency distribution of (sub-) haplotypes across the studied populations/species (The diameter of the black spots is proportional to the frequency of the haplotype identified in each population (for *H. litoreum*, the frequency was calculated based on the number of plants included in the analysis)).

The samples from Kamenjak (Croatia) showed the following sub-haplotypes: H04_b, H06_a, H06_b and six H09 sub-haplotypes (Figure 2, Figure 3 and Figure S2). The Pertusato population had only H09 sub-haplotypes and one of its individuals shared the same haplotype with the samples from Kamenjak. The population in which H09 sub-haplotypes were predominant was also from Ajaccio (75%). In other Corsican populations, H07 sub-haplotypes were dominating. Three haplotypes had a population distribution similar to H07 and were grouped in the same cluster, namely H02_a, H02_b and H11. On the other hand, eight haplotypes (H03_a, H03_b, H04_a, H04_b, H06_a, H06_b, H08 and H10) were clustered with the H09 sub-haplotypes. *H. litoreum* formed a separate group together with six samples from the Corsican population of Punta di a Vacca Morta. It is interesting to note that the only haplotype (H07_d) detected in the population of Lavu Santu was also the most widespread haplotype, present in eight populations. Haplotype H07_g was only observed in populations from the south of Corsica (Punta di a Vacca Morta, Cavu, Col de Bavella and Col de Saint Eustache). Similarly, haplotype H07_e was mostly observed in southern Corsican populations (Col de Bavella, Col de Saint Eustache and Ajaccio) with the exception of the population from Corte (center–north Corsica). Haplotypes, other than H07 and H09 (H02, H03, H04, H05, H06, H08, H09, H10 and H11) were identified only in a single or few samples. Although the majority of Corsican populations were most frequently constituted of H07 sub-haplotypes, H09 sub-haplotypes were also identified, although in minority, in almost all populations.

**Figure 4:**
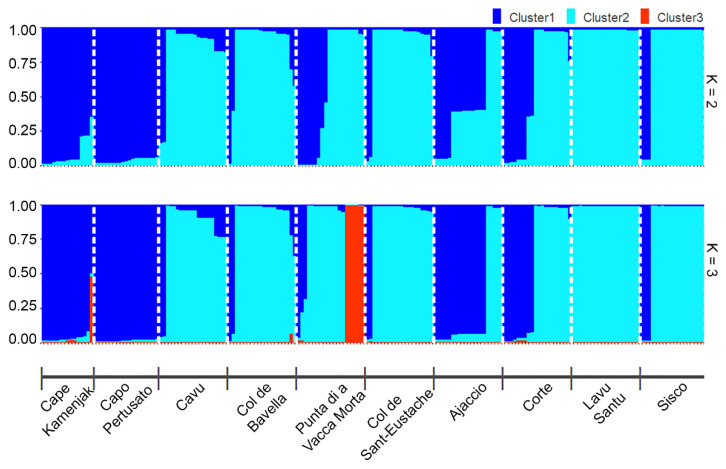
Results of the Bayesian model-based clustering STRUCTURE analysis of 194 individuals of *H. italicum*. Results were plotted when K = 2 and K = 3 based on 10 cpSSR loci.

The geographic population structure was evaluated with the Bayesian clustering method and the most likely number of genetic clusters was determined by the Evanno method [23]. When all *H. italicum* populations were analyzed, the modal value of the ΔK distribution indicated that the true K value was two (Figure 4; K = 2). Out of the ten populations studied, six (Kamenjak, Pertusato, Cavu, Col de Bavella, Col de Saint Eustache and Lavu Santu) appeared to be very homogeneous, as almost all individuals were assigned to one cluster, while the other four (Punta di a Vacca Morta, Ajaccio, Corte and, to a lesser extent, Sisco) appeared to be more heterogeneous, as individuals were assigned to two different clusters. Populations from Cape Kamenjak and Capo Pertusato were separated from the others, being mostly classified into cluster 1. Populations from Ajaccio and, to a lesser extent, Corte also had and increased number of individuals classified into cluster 1, which is more clearly shown in the plot displaying three clusters (K = 3) (Figure 4). This result is in accordance with the haplotype distribution presented in Figure 2. Although the Evanno method indicated K = 2 as the most significant K value, at K = 3 (Figure 4), we found that six samples from Punta di a Vacca Morta were defined as a third cluster, and these samples were represented by haplotypes H01 and H05. This result is comparable to evolutionary haplotype network showing the genetic similarity between the haplotypes of Punta di a Vacca Morta and the species *H. litoreum* (Figure S3). This haplotype network also demonstrated the distribution of haplotypes, similar to the distribution in the above dendrogram (Figure 3).

### 2.4. Transferability of cpSSR Markers in Helichrysum Species

**Figure 5:**
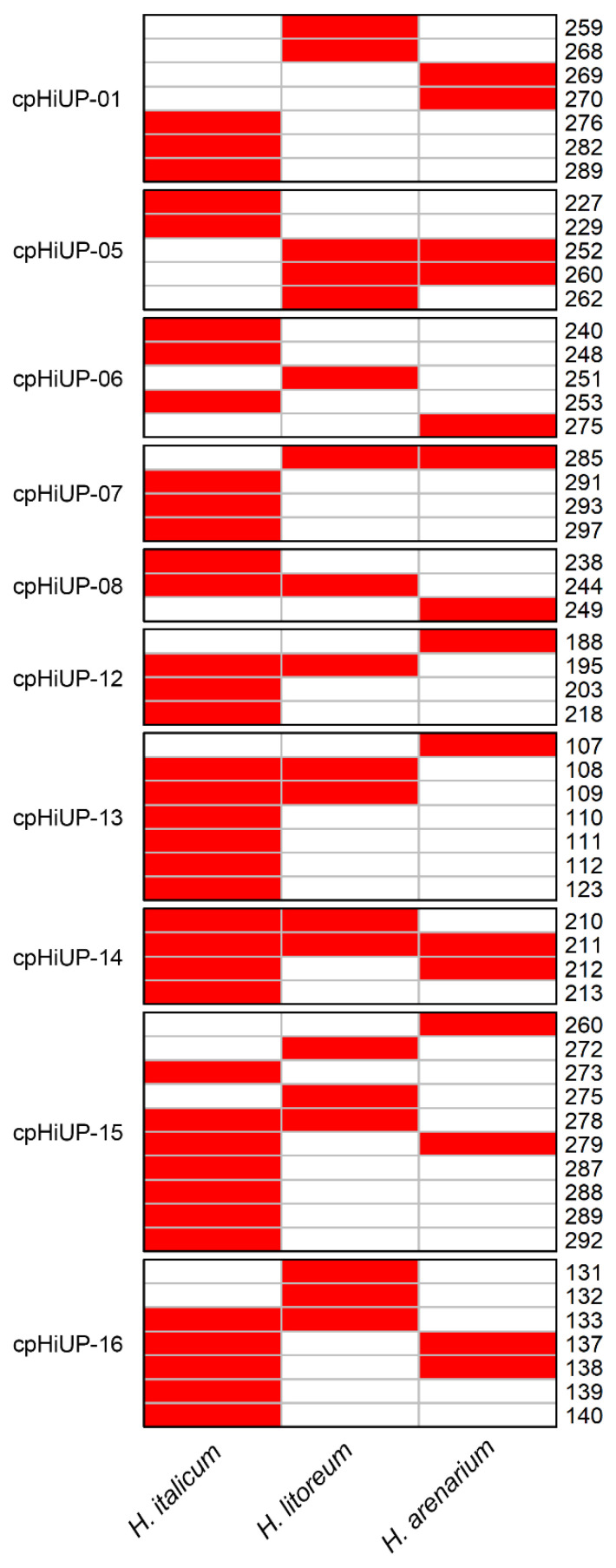
Comparison of the 56 amplified alleles across three *Helichrysum* species (*H. italicum*, *H. litoreum* and *H. arenarium*). Red color in the cells indicates the successful amplified alleles, indicated on the right side.

The new set of cpSSR markers developed from *H. italicum* was used to verify cross-species transferability with *H. litoreum* and *H. arenarium*. All 10 finally selected SSR markers showed successful amplification. Out of the 56 amplified alleles, 7 alleles were identified as private for *H. litoreum*, 6 alleles for *H. arenarium* and 26 for *H. italicum* (Figure 5). Only one allele (211) is common to the three studied species of *Helichrysum*, seven alleles are in common between *H. italicum* and *H. litoreum*, four between *H. italicum* and *H. arenarium* and three between *H. litoreum* and *H. arenarium*.

## 3. Discussion

The plant of *H. italicum* is one of the most remarkable and iconic medicinal plants of the Mediterranean area, with high morphological, genetic and chemical polymorphism in its natural populations. Currently, the essential oil of *H. italicum* is highly appreciated in the glamorous perfume and cosmetics industries for its anti-aging properties and offers great potential in the field of health care as pharmaceuticals, functional food ingredients and dietary supplements due to the unique properties of its bioactive compounds in various extracts. As a result, the commercial cultivation of *H. italicum* is increasing in many countries (France-Corsica, Portugal, Italy, Slovenia, Croatia, Serbia, Bosnia-Herzegovina, Albania and Bulgaria) [24], but no varieties with superior traits have been registered yet. The plant is only in the early stages of domestication, and cultivation in the agroecosystem is based on unverified planting material. Planting material is usually produced by the vegetative propagation of randomly selected mother plants or by seeds collected in nature, which indicates a questionable quality of the raw plant material for medicinal, pharmaceutical and cosmetic purposes. The management of planting material is therefore a huge problem, and the lack of control and certification has led to economic losses in some regions [12], mainly due to the production of essential oil with an inappropriate chemical composition or low quantities of biologically important molecules requested by the industry. Therefore, there is an urgent need for the development of molecular tools that allow the identification of local genotypes (to protect the typicality and geographical origin of local products) and the development of new varieties with higher-quality properties for the needs of the target industry, as well as strict control of the authenticity and traceability of the planting material on the market.

The chloroplast genomes of most angiosperms have a highly conserved sequence and structure, but they can also differ within species due to accumulated mutations or rare genomic rearrangements, making the chloroplast suitable for various genetic analyses. SSRs can become mutational hotspots and are therefore one of the most commonly used molecular markers. The chloroplast SSRs contain rich information on genetic variation and have been successfully used in various crop species [25,26] and in medicinal/aromatic plants [27,28,29] to study their genetic diversity, origin and population genetic structures.

In total, 43 chloroplast microsatellite regions containing mono-, di- and trinucleotide repeats were detected in this study. Comparable numbers and distributions of SSR repeats have been observed in chloroplast genomes of 12 Asteraceae species [28]. Mononucleotide repeats (A/T) were the most highly represented, as has been observed in the chloroplast genome of other higher plants [28,30,31]. Contrary to our study, tetra-, penta- and hexanucleotide repeats have also been reported in other medicinal plants from the Asteraceae family, such as *Carpesium* species [29], *Achillea millefolium* [28] and *Sinosenecio* species [32]. Also, in accordance with previous reports, most chloroplast SSRs were present in the intergenic regions [33].

Primers were successfully designed for all 43 SSRs, but a subset of 16 SSRs (including all di- and trinucleotide loci and 4 mononucleotides), selected on the basis of preliminary testing, was used to test their ability to discriminate plants within and between selected *H. italicum* populations. Based on the genotyping results, four cpSSRs (cpHiUP-02, cpHiUP-03, cpHiUP-10 and cpHiUP-11) were removed as they were either monomorphic or showed a very low level of polymorphism, while at locus cpHiUP-09, two alleles of the same length but with SNPs in the flanking region were observed (the same was observed for cpHiUP-03). The locus cpHiUP-04 was excluded due to size homoplasy (alleles with different indels but with the same final length). Indels and base substitutions in the flanking regions, which can also lead to size homoplasy, were frequently reported [22,34,35]. Insertions, consisting of duplications of several bp regions, as observed in our study, have also been previously reported in the chloroplast genome of *Citrus* species [36]. This sequence complexity could be a potential limitation in the use of cpSSR markers for estimating phylogenetic relationships in plants [21,34]. The results of the present study confirm that the sequencing of alleles of the same size in different populations or species should be performed to correctly select only the most appropriate cpSSR loci and thus reduce the presence of size homoplasy in the dataset.

Finally, 10 cpSSR markers, including six di- and trinucleotide (cpHiUP-01, cpHiUP-05, cpHiUP-06, cpHiUP-07, cpHiUP-08, cpHiUP-12) and four mononucleotide cpSSR loci (cpHiUP-13, cpHiUP-14, cpHiUP-15, cpHiUP-16), were selected as informative and suitable for the study of the plant material. A higher number of alleles per locus was observed for mononucleotide cpSSR loci than for di- and trinucleotide cpSSR loci, which is consistent with the study by Tian et al. [37] that found that mononucleotide repeats contribute more to genetic variation than other SSRs. The average values for N_a_ (3.8) and N_e_ (1.49) were slightly lower compared to the values obtained from a genotyping of 145 *Ribes* genotypes, but these were obtained from material from different species and 19 cpSSR markers were used [38]. Much higher values for both parameters (N_a_ = 8.5, N_e_ = 4.46) were obtained in another study in which 28 *H. italicum* samples from the northeastern Adriatic region were tested with genomic SSRs [12]. These observations support the fact that cpSSRs are less polymorphic than genomic SSRs due to their lower mutation rate, although the substitution rates observed in SSR loci are still higher than elsewhere in the chloroplast genome [21].

The genetic diversity and differentiation of *H. italicum* populations from different natural habitats in Corsica (France) and Cape Kamenjak (Istrian peninsula in the northeastern Adriatic, Croatia) were investigated with 10 newly developed cpSSR markers. The genetic diversity within the 10 populations was estimated using the Shannon’s information index, which provides information on the genetic variability and richness of the populations studied. In particular, the populations of Kamenjak, Punta di a Vacca Morta and Corte had the highest Shannon’s index values (I = 0.475–0.571). All other populations had lower Shannon’s index values (I = 0.169–0.268). The overall genetic variability found in this study (I = 0.309) is comparable to the 18 *H. italicum* populations from the eastern Adriatic coast (I = 0.355) analyzed with AFLP markers by Ninčević et al. [39] and by Marini et al. [40] in Tuscan Archipelago *Helichrysum* taxa (I = 0.213).

The populations of *H. italicum*, with the exception of the populations of Lavu Santu, Sisco and Ajaccio (with the lowest number of haplotypes), are characterized by high genetic diversity, which can also be observed with other statistical parameters (A, P, R_h_, H_e_). The highest values were found in the Kamenjak population, which refers to the southernmost cape of the Istrian peninsula and represents the only population of the northeastern Adriatic region in this study. The presence of rocky pastures on the Istrian peninsula, where heliophilous plants prevail, significantly enriches the plant and landscape diversity [41], which could be an explanation for its high genetic diversity. Among the Corsican *H. italicum* populations, the lowest values of genetic diversity parameters were detected in Sisco and Lavu Santu. One possible explanation for these results is that Lavu Santu is a completely isolated population originating from the beach of the same name at the mouth of the Cavu River, while Sisco represents the northernmost population of Cap Corse, which is also isolated. The other six Corsican populations (Pertrusato, Cavu, Col de Bavella, Punta di a Vacca Morta, Col de Saint-Eustache, Corte) were characterized by a high genetic diversity comparable to the Kamenjak population. A comparison of populations from the north (Haute-Corse) and the south of Corsica (Corse-du-Sud) revealed some differences in haplotype distribution. Haplotypes H9_i, H9_j, H9_h and H10 were only identified in populations from southern Corsica (Pertrusato, Col de Bavella, Col de Saint-Eustache), while haplotypes H01 and H05 were only found in the population of Punta di a Vacca Morta. Plants in this location were morphologically very different and we suspect that a natural mixture/hybridization has occurred between the two subspecies *H. italicum* spp. *italicum* and *H. italicum* spp. *thyrrenicum.* In order to gain a better insight into the genetic structure of *H. italicum* in Corsica, further populations from northern Corsica should be included in future studies along with a valid reference of *H. italicum* spp. *thyrrenicum* to control for any genetic introgression.

Two haplotypes (H07 and H09, as defined with di- and trinucleotide SSRs) were the most common in Corsica and Cape Kamenjak. The Corsican populations of Ajaccio and Pertusato and the Cape Kamenjak had predominantly H09 sub-haplotypes, while the H07 sub-haplotypes were the most prevalent at all other sites in Corsica. The result of the Ajaccio population is quite surprising, as in all other Corsican populations, with the exception of Pertusato, the H07 sub-haplotypes dominated. A possible explanation for this could be that considering the anthropogenic context of the sampled population, genetic material was introduced from other locations. Additional samples should be analyzed to confirm this hypothesis.

The presence of two clusters was confirmed by STRUCTURE analysis and clustering analysis (using Bruvo distance and Ward’s linkage method). The admixture of genetic material from two gene pools was observed in Ajaccio, Corte, Punta di a Vacca Morta and Sisco. The diversity due to differentiation (D_st_) was lower than the gene diversity within each population (H_s_ = 0.617), suggesting that most of the genetic variability was found within populations. Our results are in agreement with the studies of Ninčević et al. [39] and Marini et al. [40], who analyzed *H. italicum* populations from the eastern Adriatic region and the Tuscan Archipelago, respectively, and observed higher variability at the level of individual populations. Considering the fact that *H. italicum* is an outcrossing, entomophilous and anemochorous species [6], seeds dispersed by air flows could play an important role in its distribution and contribute to gene flow within and between populations. On the other hand, the high number of private haplotypes observed in the populations from Cape Kamenjak and Corsica could reflect the selection of specific alleles due to adaptations to ecological and environmental challenges.

All primer pairs were able to amplify the DNA of all tested *H. litoreum* and *H. arenarium* samples, indicating that the transferability rates of the new *H. italicum* cpSSR markers to other *Helichrysum* species are 100%. Furthermore, genetic analysis with cpSSRs confirmed a clear differentiation between *H. italicum*, *H. litoreum* and *H. arenarium*, and unique alleles were observed in each species. The identification of H01 and H05 haplotypes in *H. italicum* showing similarity to *H. litoreum* haplotypes indicates the usefulness of the newly developed cpSSR markers also for the possible detection of hybrids.

## 4. Materials and Methods

### 4.1. Plant Material and DNA Extraction

**Figure 6:**
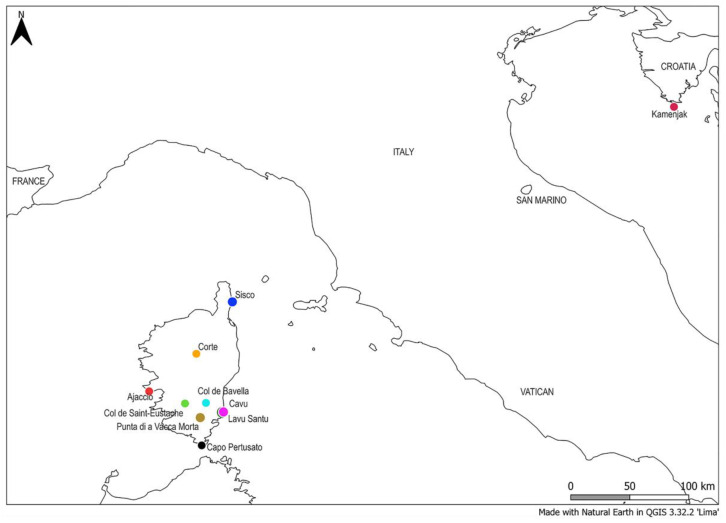
Map of *H. italicum* collection sites in Croatia (Istrian peninsula in the northeastern Adriatic region) and France (Corsica).

Populations of *H. italicum*, which can potentially serve as a source for the production of planting material, were sampled in Croatia, at the southernmost cape of the Istrian peninsula in the northeastern Adriatic region and in France (Corsica) (Figure 6, Table 6). Commercially available *H. arenarium* dry tea (purchased from Flora Ltd., Rogatec, Slovenia) and samples of *H. litoreum* obtained from certified seeds (purchased as *H. italicum* seeds, but later identified as *H. litoreum*) were included for a cross-species transferability test (Table 6). The sample from Črišnjeva was also included and served as a reference since it was previously used for the sequencing of genomic and chloroplast DNA [12,15].

To minimize potential stress or damage to the sampled individuals in the natural habitat, small tips or a few leaves were carefully removed from the plants and immediately stored in a saturated NaCl-CTAB conservation buffer [42]. Total genomic DNA was isolated using a CTAB-PVP protocol [43] with modifications, previously described by Baruca Arbeiter et al. [12]. Four separate DNA extractions were performed on randomly sampled, dried *H. arenarium* flower heads to capture genetically diverse material. DNA concentration was quantified with Qubit™ v3.0 fluorometer and Qubit™ dsDNA BR Assay Kit (Thermo Fisher Scientific, Eugene, OR, USA). DNA extracts of plants from all collection sites were deposited in the genetic laboratory of the University of Primorska (Slovenia) under the accession numbers HIUP_1-195 (*H. italicum* plants), HLUP_1-8 (*H. litoreum* plants) and HAUP_1-4 (*H. arenarium*). Sampling in Corsica was carried out under a declaration issued by the French Ministry for an Ecological and Solidary Transition (Ministère de la Transition écologique et de la Cohésion des territoires) (NOR: TREL2002508S/338).

### 4.2. Chloroplast SSR Marker Identification and Primer Development

A reference *H. italicum* chloroplast genome with GenBank accession number NC_041458.1, previously submitted by Hladnik et al. [15], served as a template for cpSSR mining. SSRs were identified using MISA v2.1 [44,45]. Misa source code was downloaded and ran on a local computer. The configuration file misa.ini was modified in order to search for microsatellites with mono- to hexanucleotide motifs. The minimum numbers of repeat units for mononucleotides, dinucleotides and higher-order repeats were 10, 5 and 5, respectively. SSRs with flanking regions 300 nt upstream and downstream were extracted from the reference genome. Extracted sequences were analyzed again with MISA tool, followed by primer design with primer3 v.2.6.1, installed via the Bioconda channel [46,47]. MISA results were piped into primer3 using a modified PERL script available at https://github.com/soltislab/transcriptome_microsats.git (accessed on 2 September 2022). Parameter PRIMER_PRODUCT_SIZE_RANGE was set to 100–300 in the p3in file. Sequences with primers were deposited in the NCBI Nucleotide database with accession numbers from PP747094 to PP747109.

### 4.3. Chloroplast SSR Marker Preliminary Testing and Genotyping

Easy scoring signals in the electropherograms corresponding to expected-size PCR products were observed in preliminary testing. Therefore, 16 newly developed primer pairs (for di-, tri- and 4 mononucleotide cpSSRs) were used for genotyping of all 207 (195 *H. italicum*, 8 *H. litoreum* and bulk samples of *H. arenarium*) samples. PCR reactions were performed using the economic method where a forward primer was elongated with M13(-21) 18 bp sequence at 5′ end for fluorescent labeling [48]. PCR reaction was performed in a final volume of 12.5 µL containing 40 ng DNA, 1× supplied AllTaq PCR buffer, 1× supplied Q-Solution, 2 mM MgCl2, dNTP mix (0.2 mM of each dNTP), 1.25 U of AllTaq DNA polymerase (Qiagen, Hilden, Germany), 0.2 mM reverse locus specific primer and M13(-21) tail labeled with fluorochrome (6-FAM, VIC, NED or PET) and 0.1 mM elongated forward primer. Locus-specific primers were synthesized by IDT-DNA and universal M13(-21) primer by Applied Biosystems (Thermo Fisher Scientific, Foster City, CA, USA).

The amplification was performed on a SimpliAmp™ Thermal Cycler (Thermo Fisher Scientific, Waltham, MA, USA). The conditions of the PCR amplification were as follows: an initial denaturation at 94 °C for 5 min, followed by 30 cycles of denaturation for 30 s at 94 °C, 45 s at the annealing temperature of 56 °C and the extension at 72 °C for 45 s. For the last 8 cycles, annealing temperature was set to 53 °C. Final extension was performed at 72 °C for 10 min [48].

A total of 1 microliter of PCR product mix, including amplicons with four different fluorescent dyes, was added to 10.7 µL Hi-Di™ Formamide (Thermo Fisher Scientific, Woolston, UK) and GeneScan™ 500 LIZ size standard (Thermo Fisher Scientific, Woolston, UK) and analyzed on a SeqStudio™ Genetic Analyzer (Thermo Fisher Scientific, Marsiling, Singapore). Electropherograms were scored with GeneMapper version 5 software (Thermo Fisher Scientific, Waltham, MA, USA).

### 4.4. Chloroplast SSR Marker Sequencing

To determine the origin of polymorphism in cpSSR loci and to confirm the nucleotide-level identity of alleles of the same length, most *H. italicum* alleles from di- and trinucleotide cpSSR loci were resequenced twice or more times. This was carried out by selecting 20 samples from different populations, each of which was sequenced across all loci. Additionally, some alleles from *H. litoreum* and *H. arenarium* were sequenced as well. An attempt was also made to sequence alleles from mononucleotide cpSSR loci, but the sequencing of some alleles failed despite several attempts.

The DNA of the selected samples was amplified as described in the “SSR amplification testing and population genotyping” section with only two differences: locus-specific primers were added in equal molar concentration (0.2 mM) and M13(-21) tail was replaced with water to the final volume of 12.5 µL per reaction. The PCR product was cleaned with Exonuclease I (Thermo Fisher Scientific, Vilnius, Lithuania) and FastAP Thermosensitive Alkaline Phosphatase (Thermo Fisher Scientific) in a final volume of 7 µL: 5 μL of PCR product, 2 U of Exonuclease I, 0.5 U of FastAP Thermosensitive Alkaline Phosphatase and 1.4 μL of 1× PCR buffer (Qiagen). The PCR cleanup reaction was incubated for 45 min at 37 °C and ended at 80 °C for 15 min. Sequencing was performed in both directions, with forward and reverse locus-specific primers, for each allele. The sequencing reaction in a total volume of 10 µL contained the following: 3.5 µL cleaned PCR product, 0.2 µL locus-specific primer (forward or reverse), 0.5 µL BigDye™ Terminator v3.1 Ready Reaction Mix (Thermo Fisher Scientific, Foster City, CA, USA), 2 µL 5× Sequencing Buffer and 3.8 µL water (Thermo Fisher Scientific). Cycle sequencing was performed on a SimpliAmp™ Thermal Cycler (Thermo Fisher Scientific) as follows: initial 3 min denaturation at 96 °C; 50 cycles with 10 s at 96 °C, 10 s at 50 °C and 4 min at 60 °C; and final incubation at 72 °C for 7 min. Sequencing reaction products were cleaned with EDTA and ethanol precipitation methods, resuspended in 12 µL Hi-Di™ Formamide (Thermo Fisher Scientific) and analyzed on a SeqStudio™ Genetic Analyzer (Thermo Fisher Scientific). Ab1 files were extracted from the Thermo Fisher Scientific cloud and analyzed with CodonCode Aligner v6.0 (CodonCode Corporation, Boston, MA, USA) to align the forward and reverse sequences.

### 4.5. Chloroplast SSR Markers Diversity Parameters, Haplotype Identification and Population Analysis

The number of alleles (N_a_), the number of effective alleles (N_e_), Shannon’s information index (I) and diversity (h) were calculated for each locus using the GenAlEx v.6.5.1 computer software (Research School of Biology, The Australian National University, Acton, Australia) [49].

The population’s genetic diversity parameters, such as the number of haplotypes (A), number of private haplotypes (P), effective number of haplotypes (N_e_), haplotypic richness (R_h_), genetic diversity (H_e_), the mean genetic distance between individuals (D^2^_sh_), relative contribution (c), gene diversity within each population (H_s_), diversity due to differentiation (D_st_), total gene diversity (H_t_) and proportion of the total genetic differentiation (F_st_) were calculated for ten *H. italicum* populations with Haplotype Analysis v.1.05 software (Forest Genetics and Forest Tree Breeding, Georg-August University Goettingen, Germany) [50].

A dendrogram with *H. italicum* and *H. litoreum* haplotypes was constructed with Bruvo’s distance [51] and Ward’s (“ward.D”) clustering method using the function bruvo.dist of the R poppr package [52] and hclust function of the stats package.

The Bayesian clustering method implemented by the software STRUCTURE [53] was used for estimating the number of populations based on chloroplast SSR data. Parameters used for the analysis were the following: length of Burnin Period 50k, number of MCMC Reps after Burnin 50k, K was set from 1K to 10K and finally 5 iterations were used. The results of STRUCTURE were subjected to the pophelper R package [54] for calculating the most likely number of genetic clusters by the Evanno method [23]. The output matrix for the best K-values was plotted using the R package pophelperShiny [54].

We also constructed an evolutionary network with haplotypes using the median-joining (MJ) method [55] implemented in the software Network 10.2.0.0. The table with the cpSSR alleles was converted to a binary matrix. As recommended by the program authors, MP calculation was run on MJ network calculations.

### 4.6. Cross-Species Transferability

The ability of the developed microsatellite markers to cross-amplify was tested in two *Helichrysum* species: *H. litoreum* and *H. arenarium*. All 12 di- and trinucleotide and 4 mononucleotide cpSSRs were amplified in both species using the same PCR protocol as described above for *H. italicum* samples. Private alleles were identified with GenAlEx for *H. litoreum* and *H. italicum*. Additionally, private haplotypes were determined for *H. litoreum* with Haplotype Analysis v.1.05. For *H. arenarium*, it was not relevant to identify haplotypes because bulk samples were used.

## 5. Conclusions

In the present study, the newly developed cpSSRs demonstrated a good discrimination ability, particularly due to the large number of private haplotypes observed in most populations, as well as the considerable genetic variation detected within populations. Additionally, due to their observed cross-species transferability, cpSSRs offer new tools for species identification, which is important for taxonomical and phylogenetic studies. These markers also show potential for identifying plant material for breeding purposes, planting material traceability at the market and conserving natural resources by studying the vitality of wild populations. The developed cpSSR markers serve as an important supplement to previously developed genomic SSR markers.

## Figures and Tables

**Table 1:** Characteristics of developed 16 cpSSR markers as observed in all *H. italicum* samples with corresponding GenBank accession numbers.

Locus	Repeat Motif	Primer Sequences (5′-3′)	Location/ Region	SSR Position	Expected Allele Size (bp)	Allele Size Range (bp)	GenBank Accession Number
cpHiUP-01	(ATT)_5_	F: *TTCATTGCACACGGCTTTCC R: TGATTTGGCCAATCATGAATGAT	*tRNK-UUU* intron	2063–2077	283	276–289	PP747094
cpHiUP-02	(TA)_5_	F: *AGGTAGTCCTTTGTGGCTGC R: TGAGACTCGAATTCCCGCTG	*RpoC1*	18669–18678	271	271	PP747095
cpHiUP-03	(AT)_5_	F: *GCCCGTAAAGGAGTTGTGGA R: TTGACAAGCCCAACCCCAAT	*RpoC2*	19665–19674	266	266	PP747096
cpHiUP-04	(AT)_5_	F: *ACCCGCCCAAAATGAAGTCA R: AGGCAAGGAGGGGAAGGATA	intergenic	36185–36194	275	253–293	PP747101
cpHiUP-05	(TA)_6_	F: *TACTTCTGGTTCCGGCGAAC R: TAGATCCGAACACTTGCCCC	intergenic	41628–41639	228	227–229	PP747102
cpHiUP-06	(AT)_6_	F: *ATTGCAAATCACGCGATCGG R: TCCCTTCTAAGCGTTCTATTTCA	intergenic	42072–42083	247	240–253	PP747103
cpHiUP-07	(TA)_5_	F: *ACGCGACATAAAGACTCCTTCT R: TCCTCTTTTCGCAAAAGCCA	intergenic	46438–46447	294	291–297	PP747104
cpHiUP-08	(TA)_5_	F: *AGATCGAAGCGAGTACCTGC R: GGCAAACGCCTACGAAAAGA	intergenic	67942–67951	244	238–244	PP747105
cpHiUP-09	(TA)_5_	F: *CTGAGACGACCCAGAAAGCA R: TTTCATCATCCGGCTCGAGC	*petD* intron	77234–77243	260	260–264	PP747106
cpHiUP-10	(AGA)_5_	F: *AGAAACTGAACAACCTCGACA R: TCGTAATGGCCTATTCCATCGG	*Ycf1*	109291–109305	215	207–213	PP747107
cpHiUP-11	(AAT)_5_	F: *TGGACAAATTATTCACAGGCCG R: TTGTAGAGTGATTCTGCGCA	*Ycf1*	111479–111493	279	274	PP747108
cpHiUP-12	(TA)_5_	F: *GAGCAGCGTGTCTACCGATT R: TCGCGAGAATTCTCTAGTTGCA	intergenic	123336–123345	202	195–218	PP747109
cpHiUP-13	(T)_13_	F: *AAGGGCTGATTTGCGGATGA R: TCCTTGCTTGCAACCCTTCT	intergenic	26467–26479	112	108–123	PP747097
cpHiUP-14	(T)_11_	F: *TTGGGGGCGATGAAACAACT R: ACGAGCAATGCCATCACCTA	intergenic	27974–27984	212	210–213	PP747098
cpHiUP-15	(A)_10_	F: *TGGACGCCTTTCATTGTGAT R: ACCCCTAGCCTTCCAAGCTA	intergenic	29779–29788	287	273–292	PP747099
cpHiUP-16	(T)_10_	F: *AGTCACTTTGGTTCCCG R: AAAGAAAGGGAAGGGGCTCGCAT	intergenic	34914–34923	134	133–140	PP747100

* Primers elongated for the universal M13 (-21) sequence (5′-TGTAAAACGACGGCCAGT-3′) at the 5′ ends.

**Table 2:** Genetic diversity parameters for the 10 polymorphic chloroplast SSR markers based on genotyping results of ten *H. italicum* populations.

Locus	N_a_	N_e_	I	h
cpHiUP-01	3	1.098	0.204	0.089
cpHiUP-05	2	1.097	0.188	0.088
cpHiUP-06	3	1.087	0.195	0.080
cpHiUP-07	3	1.975	0.772	0.494
cpHiUP-08	2	1.031	0.080	0.030
cpHiUP-12	3	1.075	0.170	0.070
cpHiUP-13	6	1.762	0.863	0.432
cpHiUP-14	4	2.053	0.913	0.513
cpHiUP-15	7	1.883	0.974	0.469
cpHiUP-16	5	1.813	0.804	0.448
Average	3.8	1.487	0.516	0.271

N_a_—number of alleles; N_e_—number of effective alleles; I—Shannon’s information index; h—diversity.

**Table 3:** Intrapopulation analysis of *H. italicum* genetic resources based on 10 chloroplast SSR markers.

Population	N	No. of alleles	No. of Private Alleles	I	A	P	N_e_	R_h_	H_e_	D^2^_sh_
Kamenjak	15	25	2	0.571	9	7	7.258	8.000	0.924	13.914
Pertusato	19	15	2	0.213	6	5	3.374	4.333	0.743	1.553
Cavu	20	15	/	0.239	5	3	4.444	3.938	0.816	0.524
Col de Bavella	20	19	1	0.268	7	3	4.000	4.998	0.789	7.319
Punta di a Vacca Morta	20	23	1	0.665	7	5	3.704	5.144	0.768	86.404
Col de Saint-Eustache	20	17	1	0.229	6	3	3.390	4.241	0.742	1.947
Ajaccio	20	15	1	0.280	4	1	2.899	2.939	0.689	30.806
Corte	20	22	2	0.457	7	4	4.878	5.421	0.837	9.962
Lavu Santu	20	10	/	0.000	1	0	1.000	0.000	0.000	0.000
Sisco	20	14	/	0.169	2	1	1.342	0.991	0.268	0.671
Mean	/	/	/	0.309	5.400	3.200	3.629	4.000	0.658	15.310

N—sample size in each *H. italicum* population; No. of alleles—number of alleles per population; No. of private alleles—number of private alleles per population; I—Shannon’s information index; A—number of different haplotypes; P—number of private haplotypes; N_e_—effective number of haplotypes; R_h_—haplotypic richness; H_e_—genetic diversity; D^2^_sh_—mean genetic distance between individuals.

**Table 4:** List of private alleles (locus name, allele length in bp and frequencies) by *H. italicum* population.

Population	Locus	Allele	Frequency
Kamenjak	cpHiUP-06	240	0.133
Kamenjak	cpHiUP-16	140	0.067
Pertusato	cpHiUP-15	279	0.053
Pertusato	cpHiUP-15	292	0.053
Col de Bavella	cpHiUP-12	218	0.050
Punta di a Vacca Morta	cpHiUP-07	297	0.300
Col de Saint-Eustache	cpHiUP-01	289	0.050
Ajaccio	cpHiUP-15	273	0.250
Corte	cpHiUP-08	238	0.150
Corte	cpHiUP-13	112	0.050

**Table 5:** Interpopulation analysis of *H. italicum* genetic resources based on 10 chloroplast SSR markers.

Population	N	c	H_s_	D_st_	H_t_	F_st_
Kamenjak	15	0.077	0.067	0.019	0.086	0.224
Pertusato	19	0.098	0.069	0.030	0.099	0.306
Cavu	20	0.103	0.080	0.022	0.102	0.217
Col de Bavella	20	0.103	0.077	0.014	0.091	0.149
Punta di a Vacca Morta	20	0.103	0.075	0.015	0.091	0.169
Col de Saint-Eustache	20	0.103	0.073	0.014	0.087	0.163
Ajaccio	20	0.103	0.068	0.029	0.096	0.298
Corte	20	0.103	0.082	0.020	0.102	0.200
Lavu Santu	20	0.103	0.000	0.034	0.034	1.000
Sisco	20	0.103	0.026	0.026	0.052	0.496
Total	194	1.000	0.617	0.223	0.840	0.266

N—sample size in each population; c—relative contribution; H_s_—gene diversity within each population; D_st_—diversity due to differentiation; H_t_—total gene diversity; F_st_—proportion of the total genetic differentiation.

**Table 6:** List of *H. italicum* populations with number of samples per population and samples of other *Helichrysum* species (*H. litoreum* and *H. arenarium*), included in the analysis.

Sampling Location	Species	Number of Samples
Croatia, Črišnjeva	*H. italicum*	1 *
Croatia, Cape Kamenjak	*H. italicum*	15
France, Corsica, Capo Pertusato	*H. italicum*	19
France, Corsica, Cavu	*H. italicum*	20
France, Corsica, Col de Bavella	*H. italicum*	20
France, Corsica, Punta di a Vacca Morta	*H. italicum*	20
France, Corsica, Col de Saint-Eustache	*H. italicum*	20
France, Corsica, Ajaccio	*H. italicum*	20
France, Corsica, Corte	*H. italicum*	20
France, Corsica, Lavu Santu	*H. italicum*	20
France, Corsica, Sisco	*H. italicum*	20
Plants from purchased certified seeds, grown on experimental collection of the University of Primorska (Slovenia) (45°34′19″ N 13°46′32″ E)	*H. litoreum*	8
Slovenia, commercially available *H. arenarium* tea (Flora Ltd., Rogatec, Slovenia)	*H. arenarium*	4 bulk samples

* A sample from Črišnjeva served as a reference for the preliminary testing of primers, as this sample had been used for the sequencing of genomic DNA and chloroplast assembly.

## Data Availability

Sequences of loci with SSRs and primers were submitted to NCBI Nucleotide database with accession numbers from PP747094 to PP747109. The original contributions presented in the study are included in the article/Supplementary Material, further inquiries can be directed to the corresponding author.

## Supplementary Materials

The following supporting information can be downloaded at https://www.mdpi.com/article/10.3390/plants13192740/s1, Table S1: Characteristics of the developed 27 mononucleotide cpSSR markers tested on four samples from different *H. italicum* populations. Figure S1: Multiple sequence alignment parts of sequenced alleles showing polymorphisms in the SSRs or flanking regions. Numbers next to polymorphisms indicate position based on reference chloroplast genome (MK089797). Figure S2: Heat table with haplotype frequencies per population. Haplotypes with bold font (from H01 to H22) were obtained with 6 di- and trinucleotide SSR markers, whereas haplotypes with underscore and a letter represent sub-haplotypes obtained with additional four mononucleotide markers. Sub-haplotypes were not identified for haplotype H01, H05, H08, H10 and H11. Six *H. litoreum* haplotypes (H12–H17) were included to highlight differences between species. Table S2: List of private sub-haplotypes with their population names and corresponding frequencies. Figure S3: Haplotype network of *H. italicum* and *H. litoreum* based on the 10 cpSSR markers.
