# Supplementary material for: New Chloroplast Microsatellites in Helichrysum italicum (Roth) G. Don: Their Characterization and Application for the Evaluation of Genetic Resources
Source: Plants (Basel). 2024 Sep 30;13(19):2740. doi: 10.3390/plants13192740 (PMC11479114; doi:10.3390/plants13192740)
Supplement: Supplementary file 1 [file plants-13-02740-s001.zip › plants-3208678-supplementary.pdf]

Supplementary data

Table S1. Characteristics of the developed 27 mononucleotide cpSSR markers tested on four samples from different *H. italicum* populations.

Locus	Repeat motif	Primer sequences (5' -3')	Location/region	SSR position	Expected size (bp)	Allele size range (bp)
cpHiUP-17	(T)11	F: ACAATTTCCGTTCTGGGGTT<br>R: GCCGAAAAACTTCCACGGAC	intergenic	8992-9265	274	203-274
cpHiUP-18	(A)10	F: GTCCGTGGAAGTTTTTCGGC<br>R: CCCGGATTTGAACTGGGGAA	intergenic	9246-9528	283	278-283
cpHiUP-19	(T)12	F: TAATGCGCCAGCCAAAATGG<br>R: GGTATTGTGGCCATTGCGTC	intergenic	12675-12790	116	112-115
cpHiUP-20	(A)10	F: GGGTTCGTGTGAGTAGGCAA<br>R: ATACCGGATCGCCCTCTACA	*RpoB*	13264-13467	204	206
cpHiUP-21	(T)10	F: TCAGAAAGAGCTCGGGACTT<br>R: GACCAATCCACCTACCCGAC	*RpoC2*	20775-21031	257	256
cpHiUP-22	(T)10	F: CAAAGAGGGGTTCGGGACAA<br>R: AAGCGGCAGAAATCAGTGGA	intergenic	25801-26080	280	279
cpHiUP-23	(A)11	F: TTCAAGACCGGGGCTTTCAA<br>R: ACATGGAGTCGGGGGAGAAT	intergenic	35106-35332	227	218-225
cpHiUP-24	(T)11	F: GATCCTACACCGCTGCTCAA<br>R: AGGGCTTTCTACATATGCATCGT	*ycf3* intron	42887-43169	283	283-290
cpHiUP-25	(A)10	F: CCCAATCACGAATCATGCGC<br>R: GGGATCATTTTCCCGCGAGA	*ycf3* intron	44102-44356	255	252-253
cpHiUP-26	(T)11	F: ACATGATAAATCGATGGCTTTTGC<br>R: TGGTCATCGGTTCGATTCCG	intergenic	46501-46642	142	141-142
cpHiUP-27	(A)11	F: TCGACATGCATAGGAATTCAAGA<br>R: TCTCTTGCACTCATTTCCCCA	intergenic	46701-46998	298	297-299
cpHiUP-28	(T)10	F: ATTTTGTTTTGTAGCCTGCTGT<br>R: TATGGCGCAACCCACTTCTT	intergenic	55129-55306	178	179
cpHiUP-29	(T)10	F: CCGGCAATCGCAATAGCTTC<br>R: TTGGGAGATTTTCGGCGGAA	intergenic	59764-59941	178	176-177
cpHiUP-30	(T)13	F: GGGCTAGAAACGAGGATCAGG<br>R: ACAACAATGTATGCATTAGTGATTCCA	intergenic	64976-65268	293	293-301
cpHiUP-31	(A)11	F: TGAGTTGGGCAATATTGGGGA<br>R: AGTACCGGTGCCACAGAAAG	intergenic	66944-67124	181	182
cpHiUP-32	(A)10	F: ACCCATAAAGGTTTGCCCGT<br>R: TGCATAATGCCCGTACGGTT	*clpP* intron	70114-70367	254	252-253
cpHiUP-33	(A)10	F: TCGCTATCAACCTCTTGGCC<br>R: GGAACACAAACTCAGGTTTCGT	*clpP* intron	71117-71341	225	223-228
cpHiUP-34	(T)11	F: GGGTAAAGATCCGCCCGATT<br>R: TGAAGTATCCAGGCTCCGTT	*clpP* intron	71538-71803	266	265-266
cpHiUP-35	(T)14	F: GGAACGGTCGCTTCAAAGTG<br>R: TGGACCTTTTGAATAACAGCCA	intergenic	77873-78154	282	277-283
cpHiUP-36	(T)13	F: TGCTACTCGAACCACGCTTT<br>R: AGTTTCATTAGCTCCCGAGGT	intergenic	80541-80832	292	292-294
cpHiUP-37	(T)11	F: GAGGCATGTTTGGAACTGCG<br>R: GCGGGTGCAGGTTTCTTTTT	intergenic	81020-81262	243	241-244
cpHiUP-38	(T)10	F: AACGTGTCACAGCTTCCTCC<br>R: GCTAGGTAAGCGCCCTGTAG	intergenic	84322-84579	258	257-258

Locus	Repeat motif	Primer sequences (5' -3')	Location/region	SSR position	Expected size (bp)	Allele size range (bp)
cpHiUP-39	(A)10	F: TCGATTGGCTAATGCTGAGACT<br>R: CCTTCCACGATACATCCCGT	*Ycf1*	109795-109921	127	127
cpHiUP-40	(A)10	F: TCCCACAAAAATGGAGAAATGGAC<br>R: AGAGGCCTTCTTCTTAGTTTCAGA	*Ycf1*	112083-112306	224	223
cpHiUP-41	(T)12	F: TGGCATGAAACAACACGCAG<br>R: TTTTGGGGCACAGGTTTTGG	intergenic	120178-120281	104	101
cpHiUP-42	(A)10	F: AATTCTTGCAACTAGAGAATTCTCGC<br>R: ACAAGTGTCAATTTGGAGTAGTCA	intergenic	123381-123556	176	176-178
cpHiUP-43	(T)10	F: ATTAGCAGCCGTTTCCAGCT<br>R: CGAACTCCGGGCGAATATGA	intergenic	136302-136572	271	272

cpHiUP-01

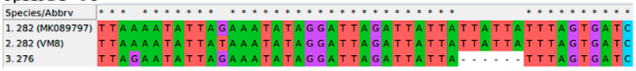

*All sequenced alleles with the same length were confirmed to be identical except in sample VM8, where one SNP (2408 A/G) was observed.

cpHiUP-03 (excluded from the analysis)

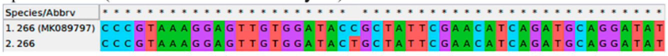

*Some of the sequenced samples had 19487 C/T mutation: CB2, CB20, CB7, CN10, CN20, VM6, VM7 and VM13.

cpHiUP-04 (excluded from the analysis)

The first excerpt of alignment with polymorphisms

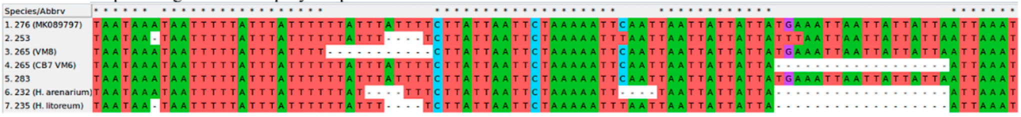

The second excerpt of alignment with polymorphisms

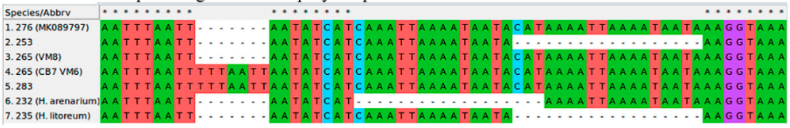

*Two alleles with the same length (265) had different nucleotide sequence.

cpHiUP-05

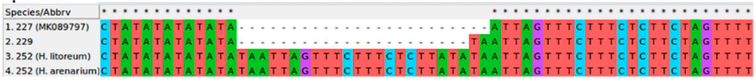

*Duplication of TAATTAGTTCTTCTCTTATA motif in allele 252.

cpHiUP-06

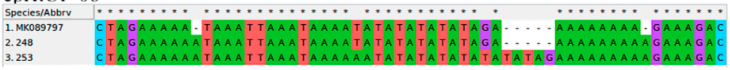

*Reference sequence (MK089797) is shorter for two bases since in the mononucleotide region wrong number of As was detected with Ion Torrent technology or error occurred during chloroplast genome assembly.

cpHiUP-07

The first excerpt of alignment with polymorphisms

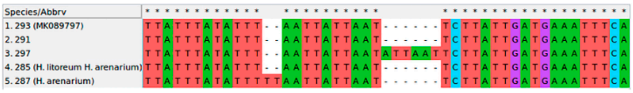

The second excerpt of alignment with polymorphisms

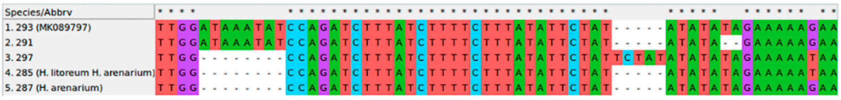

cpHiUP-08

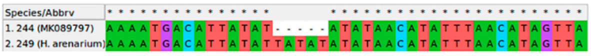

cpHiUP-09 (excluded from the analysis)

The first excerpt of alignment with a polymorphism

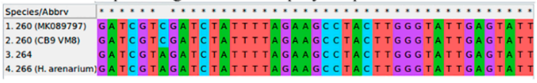

The second excerpt of alignment with polymorphisms

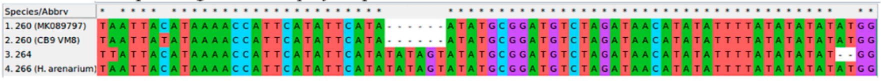

cpHiUP-12

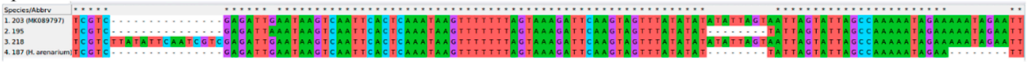

Figure S1. Multiple sequence alignment parts of sequenced alleles showing polymorphisms in the SSRs or flanking regions. Numbers next to polymorphisms indicate position based on reference chloroplast genome (MK089797).

Haplotype	Cape Kamenjak	Capo Pertusato	Cavu	Col de Bavella	Punta di a Vacca Morta	Col de Saint Eustache	Ajaccio	Corte	Lavu Santu	Sisco	*H. litoreum*
**H01**					0.20						
**H02**				0.05	0.05						
H02_a					0.05						
H02_b				0.05							
**H03**						0.05		0.05			
H03_a								0.05			
H03_b						0.05					
**H04**	0.13				0.05						
H04_a					0.05						
H04_b	0.13										
**H05**					0.10						
**H06**	0.13										
H06_a	0.07										
H06_b	0.07										
**H07**			0.90	0.85	0.60	0.85	0.25	0.55	1.00	0.85	
H07_a			0.25								
H07_b					0.05						
H07_c			0.20								
H07_d			0.15	0.40	0.45	0.45	0.10	0.15	1.00	0.85	
H07_e				0.20		0.25	0.15	0.35			
H07_f				0.05							
H07_g			0.30	0.20	0.10	0.15					
H07_h								0.05			
**H08**								0.15			
**H09**	0.73	1.00	0.10	0.05		0.05	0.75	0.25		0.15	
H09_a	0.07										
H09_b	0.07										
H09_c										0.15	
H09_d	0.07							0.10			
H09_e	0.20										
H09_f	0.13										
H09_g		0.05									
H09_h		0.32									
H09_i						0.05					
H09_j		0.11									
H09_k							0.25				
H09_l		0.42									
H09_m	0.20	0.05									
H09_n			0.10								
H09_o		0.05									
H09_p								0.15			
H09_r				0.05			0.50				
**H10**				0.05							
**H11**						0.05					
**H12**											0.13
**H13**											0.25
**H14**											0.13
**H15**											0.25
**H16**											0.13
**H17**											0.13

Figure S2. Heat-table with haplotype frequencies per population. Haplotypes with bold font (from H01 to H22) were obtained with 6 di- and trinucleotide SSR markers, whereas haplotypes with underscore and a letter represent sub-haplotypes obtained with additional four mononucleotide markers. Sub-haplotypes were not identified for haplotype H01, H05, H08, H10, and H11. Six *H. litoreum* haplotypes (H12-H17) were included to highlight differences between species.

Table S2. List of private sub-haplotypes with their population names and corresponding frequencies.

(Sub)-haplotype	Population	Frequencies
H09_k	Ajaccio	0.25
H07_a	Cavu	0.25
H07_c	Cavu	0.20
H09_n	Cavu	0.10
H02_b	Col de Bavella	0.05
H07_f	Col de Bavella	0.05
H10	Col de Bavella	0.05
H03_b	Col de Saint-Eustache	0.05
H09_i	Col de Saint-Eustache	0.05
H11	Col de Saint-Eustache	0.05
H03_a	Corte	0.05
H07_h	Corte	0.05
H08	Corte	0.15
H09_p	Corte	0.15
H04_b	Kamenjak	0.13
H06_a	Kamenjak	0.07
H06_b	Kamenjak	0.07
H09_a	Kamenjak	0.07
H09_b	Kamenjak	0.07
H09_e	Kamenjak	0.20
H09_f	Kamenjak	0.13
H09_g	Pertusato	0.05
H09_h	Pertusato	0.32
H09_j	Pertusato	0.11
H09_l	Pertusato	0.42
H09_o	Pertusato	0.05
H01	Punta di a Vacca Morta	0.20
H02_a	Punta di a Vacca Morta	0.05
H04_a	Punta di a Vacca Morta	0.05
H05	Punta di a Vacca Morta	0.10
H07_b	Punta di a Vacca Morta	0.05
H09_c	Sisco	0.15

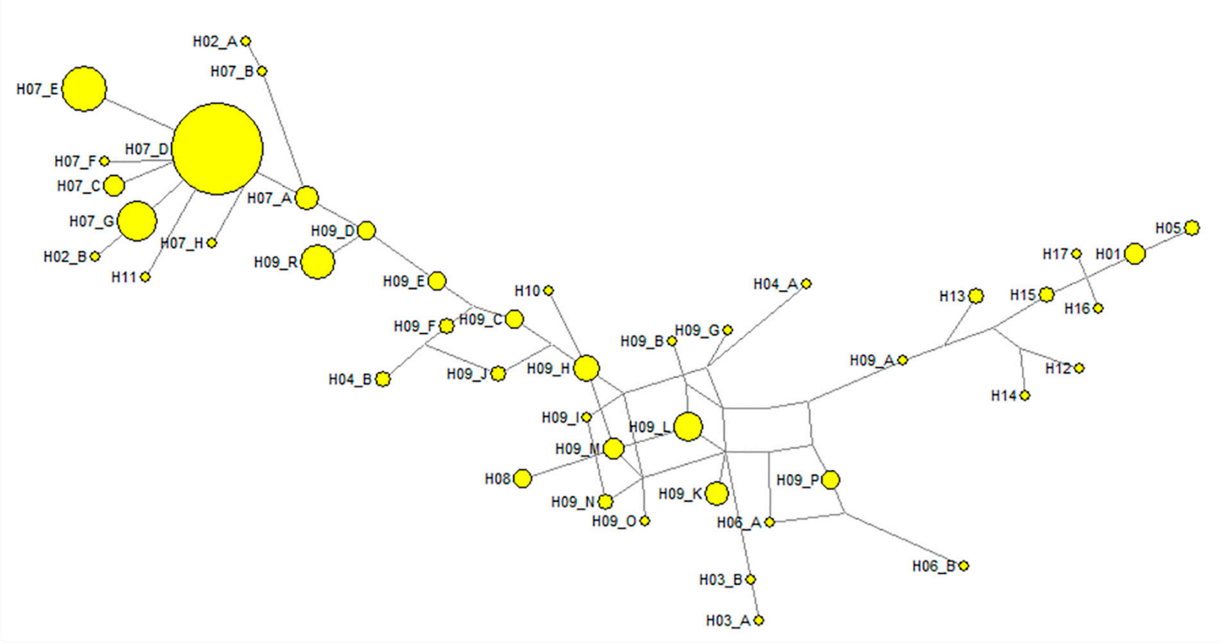

Figure S3. Haplotype network of *H. italicum* and *H. litoreum* based on the 10 cpSSR markers.
